# Improving Water Solubility and Skin Penetration of Ursolic Acid through a Nanofiber Process to Achieve Better In Vitro Anti-Breast Cancer Activity

**DOI:** 10.3390/pharmaceutics16091147

**Published:** 2024-08-29

**Authors:** Hsuan Fu, Tzu-Hui Wu, Chih-Peng Ma, Feng-Lin Yen

**Affiliations:** 1 Doctoral Degree Program in Toxicology, College of Pharmacy, Kaohsiung Medical University, Kaohsiung 80708, Taiwan; 2 Department of Pharmacy and Master Program, Collage of Pharmacy and Health Care, Tajen University, Pingtung 90741, Taiwan; u93070@tajen.edu.tw; 3 Department of Radiology, Pingtung Christian Hospital, Pingtung 90059, Taiwan; 4 Department of Medical Research, Kaohsiung Medical University Hospital, Kaohsiung 80756, Taiwan; 5 Institute of Biomedical Sciences, National Sun Yat-Sen University, Kaohsiung 80424, Taiwan; 6 Drug Development and Value Creation Research Center, Kaohsiung Medical University, Kaohsiung 80756, Taiwan; 7 Department of Fragrance and Cosmetic Science, College of Pharmacy, Kaohsiung Medical University, Kaohsiung 80756, Taiwan

**Keywords:** breast cancer, ursolic acid, nanofibers, skin absorption

## Abstract

Woman’s breast cancer has always been among the top ten causes of cancer death, and nearly 2% to 5% of locally advanced breast cancers develop a fungating breast wound. Fungal breast cancer leads to skin ulcers, wound ruptures, and other bacterial infections in patients. Ursolic acid (UA), a natural pentacyclic triterpene compound, is widely distributed in many fruits. Previous studies demonstrated that UA has anti-breast cancer, antifungal, and improved wound-healing effects. UA, however, had poor water solubility and low bioavailability, restricting its clinical application. Nanofibers have the advantages of rapid dissolution, improved stability, and bioavailability of active ingredients. We had successfully prepared ursolic acid nanofibers (UANFs) and effectively improved their water solubility and skin penetration. UANFs can increase water solubility by improving the physicochemical properties, including increased surface area, intermolecular bonding with excipients, and amorphous transformation. Furthermore, UANFs had better anti-breast cancer activity than raw UA. UANFs inhibited the expression of phospho-signal transducer and activator of transcription 3 (STAT3) and phospho-extracellular regulated protein kinases (ERK)1/2, and induced cleaved caspase-3 protein expression, but had no effect on the raw UA treatment. In summary, UANFs enhanced the skin absorption of UA and improved its anti-breast cancer efficacy. We expect that UANFs can be used as an anti-breast cancer treatment and reduce the discomfort of breast cancer patients during dressing changes, but more detailed efficacy and safety trials still need to be conducted in further studies.

## 1. Introduction

Breast cancer, a type of cancer that is commonly diagnosed in women worldwide, is the second leading cause of cancer-related deaths in females. It is estimated that around 2.3 million women have been diagnosed with breast cancer, resulting in a total of 670,000 deaths. Breast cancer incidence and mortality rates are particularly high in developed countries. However, survival from breast cancer is widely inequitable between and within countries; nearly 80% of mortalities from breast cancer occur in low- and middle-income countries [1,2,3]. Breast cancer is typically treated with surgical resection, followed by chemotherapy, radiotherapy, or a combination of both [4]. Nevertheless, both treatment methods may cause adverse reactions. New treatment methods such as targeted therapy and immunotherapy offer fewer adverse reactions, yet are costly [5]. In addition, breast cancer is unlike other types of cancer, since it may cause the formation of fungating tumors [6]. A fungating tumor usually occurs when lesions become serious and the cancer cells start to penetrate the area of skin that surrounds the breast. The penetration of cancer cells into the skin causes ulcers, a break or discontinuity of the skin, as well as infections [7,8]. Therefore, we expect that a good anti-breast cancer treatment should have the following characteristics: (1) possess a reduced amount of side effects; (2) possess antifungal effects; (3) possess wound healing effects; and (4) possess an affordable price.

Various natural plant-derived products have been suggested as alternative treatments for different types of cancer. Compared with chemotherapy or radiotherapy, plant extracts or natural compounds are less toxic, resulting in fewer side effects of treatment. Plant extracts such as *Allium sativum*, *Curcuma longa*, and *Arctium lappa* all have anti-breast cancer effects. Although natural therapy cannot completely cure breast cancer, it can be used as an auxiliary treatment in conjunction with other treatments. Ursolic acid (UA, 3 β-hydroxy-urs-12-en-28-oic acid) is a natural arbutin pentacyclic triterpenoid compound that is widely found in various spices (such as rosemary, thyme), fruits (such as apples, cranberries) and medicinal materials (such as loquat). It has been proven to be capable of regulating cancer cells through various mechanisms and signaling pathways [9,10]. UA also effectively promotes breast cancer cell apoptosis, induces cell cycle arrest, inhibits tumor metastasis, and can be used as an adjuvant chemotherapeutic candidate [11,12,13,14,15]. Furthermore, UA possesses anti-inflammatory, antifungal, and wound-healing properties [16,17]. To be concise, UA has numerous biological activities and has attracted much attention as a possible natural anticancer treatment.

Unfortunately, UA has low water solubility, poor absorption of oral drugs, a short half-life, and low bioavailability; therefore, Yu D et al. classified UA as an active ingredient in Category IV according to the Biopharmaceutical Classification System (BCS) [18]. Over the past few decades, researchers have utilized nanotechnologies, such as lipid vesicles [19], nanocrystals [20], and nanoparticles [21], to improve the solubility and absorption of UA, thereby promoting breast cancer cell apoptosis or inhibiting cell proliferation to increase anti-cancer activity. However, there are still some concerns regarding the use of these dosage forms in large-area fungal breast ulcers. For example, liquid preservation requires the use of preservatives, and organic solvents are not conducive to mass production by manufacturers. Nanofibers, forming a preservative-free dry drug-delivery system through an electrospinning process, enable active ingredients to be carried onto high-molecular polymers which will quickly dissolve and cause an increase in absorption. Additionally, nanofibers can be directly employed in patients with extensive lesions from breast cancer to reduce discomfort during topical administration. The materials that were used to prepare nanofibers in this study include 2-hydroxypropyl-β-cyclodextrin (HPBCD) and polyvinylpyrrolidone (PVPK90), both of which are approved by the Food and Drug Administration (FDA) as highly safe drug carriers and are frequently used in improving the absorption of compounds which have poor water solubility.

Hence, this study focuses on the preparation of ursolic acid nanofibers (UANFs), and explores their anti-breast-cancer activity. It is anticipated to promote the absorption of ursolic acid to boost its efficacy while producing a fast-dissolving, preservative-free UANF. The present study aims to analyze the physicochemical properties, in vitro skin absorption, and protein expressions of apoptosis in breast cancer cells to compare the anticancer activity of raw UA and UANFs. Hence, we anticipate that UANFs could be developed as an auxiliary medication to treat fungating breast tumors. This will offer an alternative viewpoint on surgical breast cancer treatment while also reducing the risk of infection from major surgical wounds.

## 2. Materials and Methods

### 2.1. Preparation of Ursolic Acid Nanofibers (UANF)

The electrospun nanofiber was woven by FES-COS Electrospinning equipment (Falco Tech Enterprise Co., Taipei, Taiwan). Briefly, 25 mg of UA (Changsha Staherb Natural Ingredients Co., Ltd., Changsha, China) and different ratios of hydroxy-pro-pyl-beta-cyclodextrin (HPBCD, Zibo Qianhui Biological Technology Co., Ltd., Zibo, China) were dissolved in 5 mL of methanol and stirred for 1 h. Then, polyvinyl pyrrolidone (Luviskol^®^ K90 Pow-der, PVP, Wei Ming Pharmaceutical Mfg. Co., Ltd., Taipei, Taiwan) was added afterward, and the solution was stirred for 1 h to obtain the electrospinning solution. Next, transfer the solution to a plastic syringe with a 0.22 mm internal diameter needle. The conditions of the electrospinning process are as follows: the tip–collector distance was 10 cm, the injection rate was set at 0.02 mL/h, and the voltage was set at 12 KV. During the process, nanofibers were formed and collected on the collector plate with aluminum foil.

### 2.2. High-Performance Liquid Chromatography (HPLC) Analysis of UA and UANF

The calibration curve of UA and the content of UA in UANFs were analyzed by HPLC method. Using 0.1% glacial acetic acid and methanol solution (5:95; *v*/*v*) as the mobile phase, the flow rate was 1 mL/min. The analysis column was the Mightysil RP-18 GP column (250 × 4.6 mm i.d., 5 μm) and the wavelength of the UV detector was 215 nm. After HPLC analysis, the retention time of UA displayed at 3.5 min and the calibration curve concentration range of UA was 0.5–100 μg/mL, and showed a great linear relationship (r > 0.999).

### 2.3. Observation of Diameter and Morphology of UANF

Using an ion-coater (E-1045, Hitachi, Tokyo, Japan) to coat each sample with platinum, the power was set to 10 mA with a time length of 120 s. The morphology and structure were observed with a scanning electron microscope (SEM, Hitachi S4700, Hitachi, Tokyo, Japan), and then the diameter of the nanofibers in the SEM images was analyzed using ImageJ software 1.53 v.

### 2.4. Particle Size Measurement of UANFs

After being dissolved in water, the particle size of various nanofibers was measured by a particle size analyzer ELSZ-2000 (Otsuka Electronics, Osaka, Japan) using dynamic light scattering (DLS) technology. Then, the uniformity of the UANF morphology was detected using a transmission electron microscope (TEM, JEM-2000EXII instrument, JEOL Co., Tokyo, Japan). The samples were dropped into the copper mesh, then added 0.5% (*w*/*v*) phosphotungstic acid. Samples were observed by the TEM after drying.

### 2.5. Drug Loading of UANF

To detect the drug loading of UANFs, 1 mg of the nanofibers were dissolved in 1 mL of methanol, and the solution was analyzed by the HPLC method to measure the concentration of UA. The following formula was used to calculate drug loading:

Drug loading (%) = (C_UA_ × V_UANF_)/W_UA_ × 100% (1)

where C_UA_ is the concentration of UA from UANFs, V_UANF_ is the volume of UANF solution, and W_UA_ is the theoretical amount of UA added.

### 2.6. Encapsulation Efficiency of UANFs

The encapsulation efficiency indicated whether the nanofibers successfully encapsulated the active compounds so that they could be dissolved in the solution to form nanoparticles. UANF was dissolved in deionized water, which was then transferred to a centrifugal filter device (Microcon YM-10, Millipore, Billerica, MA, USA). Next, a refrigerated centrifuge (Centrifuge 5430R, Eppendorf, Hamburg, Germany) was used to centrifuge the centrifugal filter devices at 12,000 rpm for 10 min. Due to the difference in molecular weight, the upper tube retained the encapsulated part (molecular weight greater than 1 × 10^4^), and the lower tube collected the unencapsulated part (molecular weight less than 1 × 10^4^). The solution of the unencapsulated part was analyzed using the HPLC method. The formula was used to calculate the encapsulation efficiency:

Encapsulation efficiency (%) = (A_UA_ − A_unentrapped UA_)/A_UA_ × 100% (2)

where A_UA_ is the theoretical amount of UA incorporated into the nanofibers, and A_unentrapped UA_ is the amount of unencapsulated UA.

### 2.7. Water Solubility of UANFs

Pure UA and UANFs were respectively dissolved in deionized water, and sonicated by the ultrasonicator for 20 min. Then, the samples were filtered using a 0.45 μm filter (Pall Corporation, Washington, NY, USA) and then analyzed using the HPLC method above.

### 2.8. Determination of Crystalline-to-Amorphous Transformation

The crystalline form of pure UA, excipients, and UANFs were analyzed with X-ray diffractometry (XtaLAB Synergy-DW, Rigaku, Tokyo, Japan). The analysis was measured using nickel-filtered Cu-Kα radiation at 25 mA and 40 kV. Its scanning angle range was from 5° to 50°, and the scan rate was 1°/min.

### 2.9. Chemical Structure of UANFs Analyzed by Fourier-Transform Infrared (FTIR) Spectroscopy

Potassium bromide (KBr) was incorporated into the samples and hard-pressed into tablets. Analysis was then performed using an FTIR spectrophotometer (Perkin-Elmer 200 Spectrophotometer, Perkin-Elmer, Norwalk, CT, USA) with a scanning range of 400–4000 cm^−1^.

### 2.10. Chemical Structure of UANF Analyzed by Nuclear Magnetic Resonance (NMR) Spectroscopy

Excipients, UA and different ratios of UANFs were dissolved in 99.8% DMSO-*d*6 (Merck, St. Louis, MO, USA), and analyzed using a JEOL Alpha 400 spectrometer (Nihon Denshi Co., Tokyo, Japan).

### 2.11. Ex Vivo Skin Penetration of UA and UANFs

This step of the experiment was carried out using a modified methodology from the European Cosmetic Toiletry and Perfumery Association’s (COLIPA) guideline standard protocol [22]. The Franz diffusion cell system was divided into two parts, including the receptor chamber and the donor chamber. Pig skin was purchased from the local butcher. The Franz diffusion cell was kept at 32 °C. Firstly, buffer solution, which comprised 0.14 M NaCl, 2 mM K_2_HPO_4_, and 0.4 mM KH_2_PO_4_ (pH 7.4), was added to the receptor chamber, and the buffer was stirred by a magnetic bar at 600 rpm during the testing. Secondly, we placed pig skin between the two chambers, dermal-side down. Into the donor chamber, the theoretical content of 200 μg of UA or UANF was added. After the pig skin had been treated with the sample for 1 h, 2 h, or 4 h, respectively, the pig skins were removed. The pig skin was striped 15 times by tape, to obtain the stratum corneum. Then, the residual skins were heated to 95 °C and separated into the epidermis and dermis with a scalpel. Each part of the pig skin was soaked in methanol and sonicated for 1 h. The content of UA in each part of pig skin was analyzed using the HPLC method.

### 2.12. Cytotoxicity

Human breast cancer MCF-7 cells were kindly provided by assistant professor Chien-Ju Lin (Kaohsiung Medical University, Kaohsiung, Taiwan). MCF-7 cells were cultured with DMEM medium (Himedia Laboratories, Mumbai, India) containing 1% penicillin-streptomycin (Biological Industries, Connecticut, NE, USA) and 10% fetal bovine serum (Hazelton Product, Denver, PA, USA), in a 37 °C incubator (Thermo Fisher Scientific, Waltham, MA, USA) with 5% CO_2_. A total of 1 × 10^4^ cells of MCF-7 was added to each well in a 96-well plate and cultured for 24 h. Then, the culture medium was removed and added to different concentrations of samples in the treatment medium. The fetal bovine serum was removed from the treatment medium, to avoid the cells from affecting the absorption of the samples. After 24 h, 0.5% of MTT (3-(4,5-cimethylthiazol-2-yl)-2,5-diphenyl tetrazolium bromide, MDBio, Taipei, Taiwan) solution was added to the plate for 3 h. Then, the absorbance was measured by a spectrophotometer (BioTek μQuant, Winooski, VT, USA) at 550 nm. The following formula was used to calculate cell viability:

Cell viability (%) = OD sample/OD control × 100%(3)

### 2.13. Anti-Breast Cancer Activity Assay by Western Blot Analysis

The anti-breast cancer effect of UA and UANFs in MCF-7 cells were analyzed using the Western blotting method. Firstly, 4 × 10^5^ cells of MCF-7 were seeded in 6-well plates and cultured in the incubator for 24 h. Then, MCF-7 cells were treated with UA or UANF in the treatment medium at various concentrations for various time lengths. After that, the proteins from the cells were lysed with RIPA Lysis Buffer (Merck Millipore, Burlington, MA, USA), and then collected into microcentrifuge tubes. The BCA protein assay kit (Thermo Fisher Scientific, Waltham, MA, USA) was used to determine the protein concentrations. To separate proteins, equal amounts of protein were loaded onto sodium dodecyl sulfate–polyacrylamide gel electrophoresis (SDS-PAGE), and then blotted onto polyvinylidene difluoride (PVDF) membranes. Before being incubated with primary antibodies overnight at 4 °C, the membranes were blocked and washed. Primary antibodies included phospho-signal transducer and activator of transcription 3 (STAT3) (Cell Signaling Technology, Danvers, MA, USA), GAPDH, p38, and extracellular regulated protein kinases (ERK) (Santa Cruz Biotechnology, Dallas, TX, USA), phospho-p38α and phospho-ERK (Merck Millipore, Burlington, MA, USA), tumor necrosis factor (TNF)-α and cleaved caspase-3 (ABclonal Technology, Woburn, MA, USA). Internal controls were the antibodies against GAPDH. Then, the membranes were incubated with secondary antibodies. After that, the membranes were reacted with enhanced chemiluminescence reagents (ECL; Thermo Fisher Scientific). Using Touch Imager (e-BLOT, Shanghai, China) to visualize protein signaling bands, and the expression of bands was measured with ImageJ software 1.53 v.

### 2.14. Statistical Analysis

Data were presented as mean ± standard deviation (SD). The statistical significance of the experimental group was performed using analysis of variance (ANOVA) with a post hoc Tukey’s test. *p* < 0.05 indicates statistical significance.

## 3. Results

### 3.1. Surface Morphology of Excipients, Ursolic Acid and Its Nanofibers

**Figure 1:**
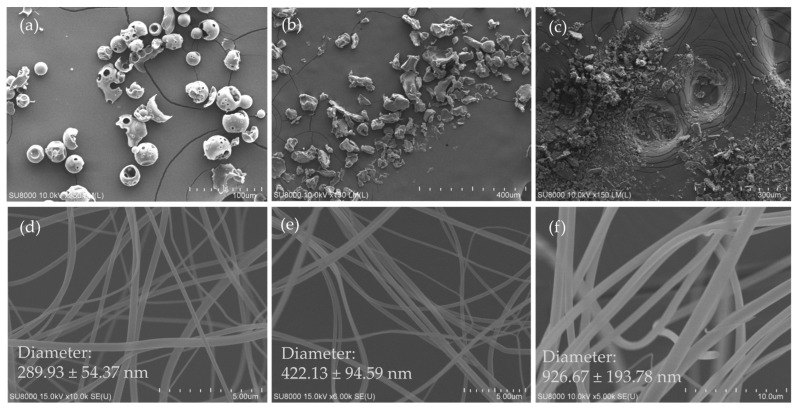
Scanning electron microscope (SEM) photographs showing the surface morphology of excipients, UA and its nanofibers. (**a**) HBPCD, (**b**) PVPK90, (**c**) UA, and the different ratio of UANFs (UA: PVP: HPBCD, *W*/*W*/*W*) (**d**) 1:8:10, (**e**) 1:8:20, (**f**) 1:8:40.

The appearance of HPBCD was sphere-shaped and porous (Figure 1a), PVPK90 was an irregular polygonal particle (Figure 1b), and their particle sizes were about 50 μm. UA was viewed as a grainy powder with a size of 2–50 μm (Figure 1c). The diameter of different ratios of UA nanofibers, and their mean fiber diameters were 289.93 ± 54.37 nm, 422.13 ± 94.59 nm, and 926.67 ± 193.78 nm, respectively (Figure 1d–f). These findings specify that the HPBCD and PVPK90 ratio have a dose-dependent effect on the fiber diameter of UANFs.

### 3.2. Powder X-ray Diffraction Pattern of Ursolic Acid and Its Nanofibers

**Figure 2:**
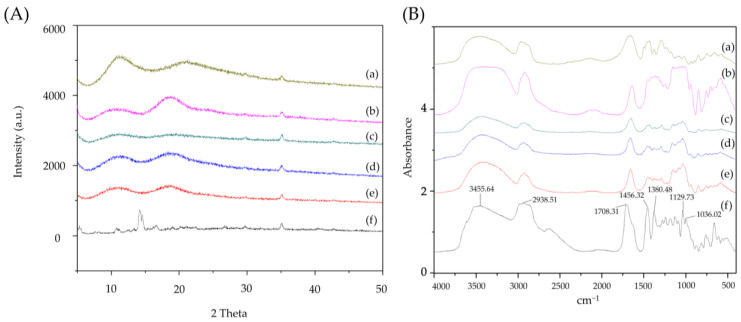
XRD spectra (**A**) and FTIR spectra (**B**) of excipients, ursolic acid and UANFs with different ratios. (a) PVPK90, (b) HPBCD, the different ratio of UANFs (UA:PVP:HPBCD, *W*/*W*/*W*) (c) 1:8:10, (d) 1:8:20, (e) 1:8:40, and (f) ursolic acid.

At a scanning angle of 13°–15°, pure UA exhibited multiple high-intensity characteristic diffraction peaks (Figure 2A). This result suggests that UA is a crystalline compound which showed low solubility in water. The XRD patterns of HPBCD and PVPK90 show where two halo broad peaks can be observed. These results show that both excipients are amorphous compounds and easily dissolve in water. On the contrary, all characteristic diffraction peaks of UA in UANFs disappeared, and similar halo patterns of HPBCD and PVPK90 were found in UANFs. These results indicate that UA was transformed to an amorphous nature after electrospinning processing and UANFs were dissolved in water with ease; therefore, we could assume that UA was successfully encapsulated by excipients in UANFs.

### 3.3. FTIR Spectra of Ursolic Acid and Its Nanofibers

FTIR spectra of UA and UANFs, with different ratios and excipients are illustrated in Figure 2B. The FTIR spectrum showed that UA had several chemical functional groups, including the broad and intense absorption band at 3455 cm^−1^ (corresponding to OH functional groups), 1708 cm^−1^ (corresponding to C=O functional groups), and 1456 cm^−1^ (corresponding to CH_2_ functional groups). The absorbance of these functional groups shifted to lower wavelengths, when UA complexed with excipients. The shift demonstrates the intermolecular hydrogen bonding formation between UA, PVPK90 and HPBCD. From these observations, it can be concluded that UA had formed new intermolecular bonds, and had been encapsulated by nanofibers.

### 3.4. ^1^H NMR Spectra of Ursolic Acid and Its Nanofibers

**Figure 3:**
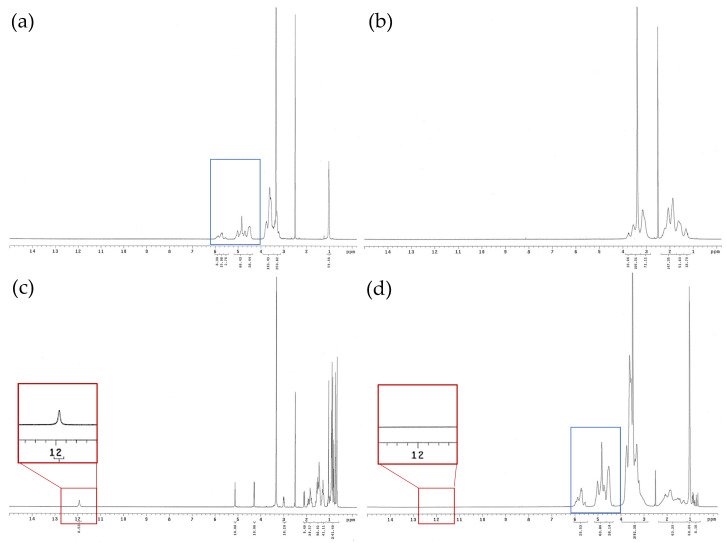
NMR spectra of ursolic acid, UANFs with different ratios and excipients. (**a**) HPBCD, (**b**) PVPK90, (**c**) ursolic acid, and (**d**) the UANFs (1:8:40). Red squares indicated the UANF spectrum did not show the carboxyl signal and blue squares indicated the signals in the UANF spectrum at 4 to 6 ppm also have similar behavior to the signals in the excipient spectrum.

In order to evaluate the hydrogen bond formation between UA and excipients, 1H-NMR analysis spectrum was used to confirm this. The ^1^H NMR spectrum of UA exhibited methyl protons (δ1 ppm), a hydroxy proton signal at δ3.33 ppm (H3), double-bound protons at δ5.12 ppm (H12), and a carboxyl signal at δ12 ppm (H28), shown in Figure 3c. However, the chemical shifts of methyl, hydroxy, and double-bound protons were moved upfield in the UANF spectrum (Figure 3d). In addition, the spectrums of excipients did not show the carboxyl signal of UA, and the UANF spectrum did not show the carboxyl signal either (red squares). Moreover, the signals in the UANF spectrum at 4 to 6 ppm also have similar behavior to the signals in the excipient spectrum (blue squares). These results proved the construction of intermolecular hydrogen bonds between UA, HPBCD and PVPK90, which indicated successful encapsulation of UANFs.

### 3.5. Drug Loading, Water Solubility, Encapsulation Efficiency, and Particle Size of Ursolic Acid Nanofibers

UANFs with the lower HPBCD ratio (1:8:10) had a better drug loading percentage than the nanofibers with the higher HPBCD ratio (1:8:20 and 1:8:40). The encapsulation efficiency of these nanofiber formulations was higher than 99%. This finding demonstrated that excipients effectually encapsulated UA after the electrospinning process. Furthermore, the raw ursolic acid’s water solubility cannot be determined, since it is below the HPLC method’s detection limit of 0.01 µg/mL. In contrast, the water solubility of UANFs showed that the increase in HPBCD content intensely improved the water solubility of pure ursolic acid. Compared with UA, the water solubility of 1:8:40 UANF increases by more than 1000-fold.

### 3.6. Particle Size and Morphology of UANFs Reconstituted in Water

**Figure 4:**
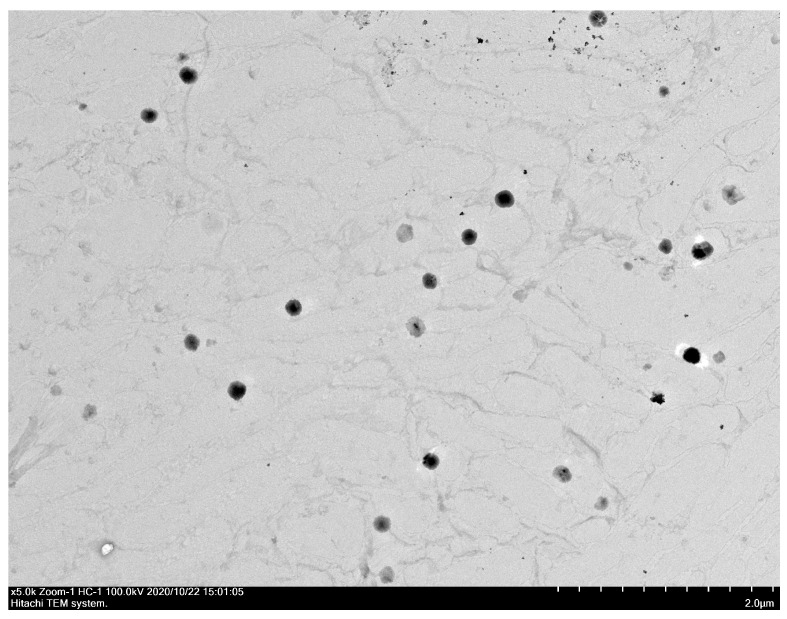
Transmission electron microscope (TEM) photograph of ursolic acid nanofibers (UANFs 1:8:40).

A laser particle-size analyzer and TEM were used to observe the particle size and appearance of the UA and its nanofiber (Table 1). The particle size and polydispersity index (PDI) of UANFs were 258.87 ± 14.81 nm and 0.29 ± 0.04, respectively. The PDI of UANFs was less than 0.3, which indicates uniform particle distribution. In contrast, UA had a bigger particle size (3370.00 ± 320.07 nm) than UANFs and displayed multi-dispersion (PDI = 1.43 ± 0.12). Moreover, the TEM photograph of UANFs reconstituted in water displayed about 200 nm spherical particles uniformly (Figure 4). These results indicate that the electrospinning process caused a reduction in the particle size of UA and enhanced its surface area.

### 3.7. In Vitro Skin Penetration of Ursolic Acid and Its Nanofibers

**Figure 5:**
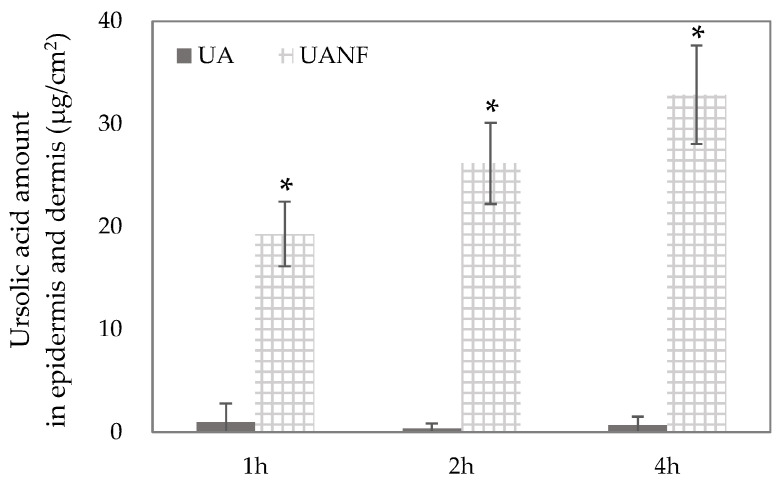
The skin penetration of pure ursolic acid (UA) and ursolic acid nanofibers (UANFs 1:8:40) in porcine skin was observed at different times using an in vitro Franz diffusion cell. Results are shown as mean ± SD (n = 5). * *p* < 0.05 significance with raw ursolic acid.

As shown in Figure 5, it was found that raw UA had very little skin penetration. After 1, 2, and 4 h of topical administration, the low content of UA (<5 µg/cm^2^) was detected in the epidermis and dermis. Compared to raw UA, the UANFs intensely increased the content of UA in the epidermis and dermis, with 19.29 µg/cm^2^, 26.17 µg/cm^2^ and 32.86 µg/cm^2^ after 1, 2, and 4 h topical administration, respectively. The findings imply that topical administration of UANFs can assist UA in penetrating into deep skin layers (*p* < 0.05), and that UANFs could penetrate skin in a time-dependent manner. Therefore, the 1:8:40 UANFs can be used to determine anti-breast cancer activity in the human breast cancer cell line MCF-7 model.

### 3.8. Ursolic Acid Nanofibers Showed Better Anti-Breast-Cancer Activity than Raw Ursolic Acid

#### 3.8.1. UANFs Significantly Inhibit Growth of MCF-7 Cells

**Figure 6:**
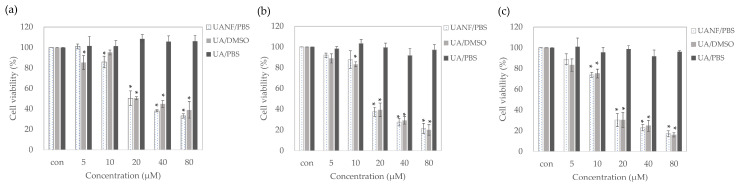
The human breast cancer cell MCF-7 was incubated with ursolic acid (UA) and its nanofiber (UANF) for (**a**) 24 h, (**b**) 48 h and (**c**) 72 h, and then the cell viability was determined by MTT assay. UA was dissolved in DMSO or PBS, and the UANF was dissolved in PBS. Results are shown as mean ± SD (n = 3). * *p* < 0.05 significance with ursolic acid dissolved in PBS.

The raw UA could be completely dissolved in DMSO, which has significant inhibition for MCF-7 cells. After 20 μM UANF/DMSO treatment for 24 h, MCF-7 cells were semi-cytotoxic, while at 48 h and 72 h, the survival rate of MCF-7 was less than 40%. However, UA has poor water solubility, and cannot be dissolved in PBS well enough to inhibit the growth of MCF-7 cells. As stated in the above results, UANF could increase the water solubility of UA, and it has a comparable efficacy in decreasing breast cancer cells to that of UA dissolved in DMSO, at all time points (Figure 6). Subsequent investigations of UANFs’ anticancer activity were conducted using a concentration of 20 μM, which significantly inhibited the growth of MCF7 at all time points.

#### 3.8.2. UANFs Have Anti-Breast Cancer Expression in MCF-7 Cells, Using Western Blot

**Figure 7:**
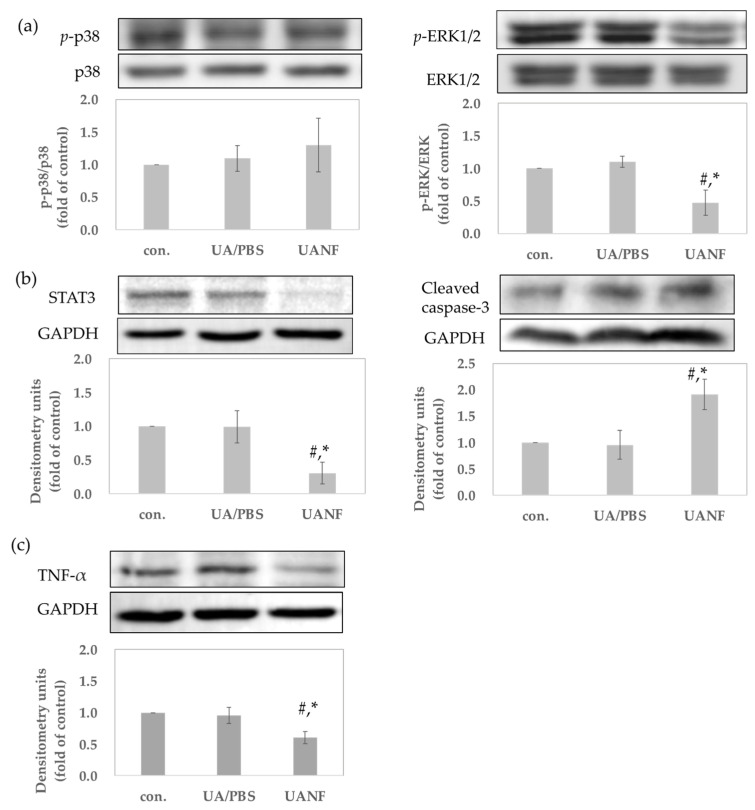
Ursolic acid (UA) and its nanofiber (UANF) affected the protein expression related to anti-breast cancer in MCF-7 cells. (**a**) Phosphorylation of p38 and ERK1/2, and the protein expression of (**b**) STAT3, cleaved caspase-3, and (**c**) TNF-α. # *p* < 0.05 significance with negative control. * *p* < 0.05 significance with UA/PBS.

To determine whether UANFs possess anti-breast cancer effects, as in previous studies of UA, we treated UA and UANFs in PBS with human breast cancer cells MCF-7 for 24 h followed by a Western blotting examination to verify the protein expression. Mitogen-activated protein kinase (MAPK) regulates cell proliferation, gene expression, differentiation, mitosis, cell survival, and apoptosis [23]. As it is related to anti-cancer performance, the phosphorylation of p38 and extracellular-regulated kinase 1/2 (ERK1/2) were tested. Figure 7a showed that raw UA in PBS could not downregulate the MPAK phosphorylation. In contrast, UANFs significantly diminished the expression of p-ERK1/2 (*p* < 0.05) but had no effect on p-p38. Furthermore, UANFs greatly decreased the expression of STAT3 and increased the expression of cleaved caspase-3 (*p* < 0.05) (Figure 7b). STAT3 is an important transcription factor, whose degradation and inhibition cause increased apoptosis. Additionally, increased cleaved caspase-3 signifies cancer cells’ apoptosis. Therefore, we suggested that UANFs could increase the apoptosis in MCF-7 cells. The present study also indicated that over-expression of TNF-α increased the metastatic activity of tumor lines [24,25]. UANFs could decrease the expression of TNF-α (*p* < 0.05), and UA did not express enough activity in MCF-7 cells (Figure 7c). These outcomes supported the fact that UANFs had better anti-breast cancer activity when compared to raw UA.

## 4. Discussion

UA has a variety of activities, such as anti-inflammatory, antioxidant, anti-allergic, antiviral, antibacterial, hepatoprotective, etc. Moreover, it possesses inhibitory effects on various cancers [26] and is a high-profile natural anti-cancer treatment. UA is highly effective in fighting breast cancer. The current pharmacological mechanism of UA against breast cancer works mainly through the following: the down-regulation of cyclin D1, STAT3, and EGFR, inducing cell cycle and growth arrest [27]; the activation of caspase-9 and caspase-3 through the mitochondrial death pathway to induce apoptosis in MDA-MB-231 breast cancer cells [28,29]; the suppression of p-PI3K, p-AKT, and p-ERK; and enhanced p-FoxO1/FoxO3a expression to significantly decrease migration [30]. Therefore, we learned that UA can inhibit the growth of breast cancer through inducing autophagy and apoptosis, inhibiting inflammatory responses, as well as inhibiting proliferation [12].

Nevertheless, a subset of women with locally advanced breast cancer presented with a fungating tumor mass eroding and infiltrating the surrounding breast skin. These patients often experience chronic pain, large open wounds, frequent infections, and malodorous drainage [7]. To address these challenges, UA can be utilized, given its antifungal and wound-healing effects. Weng et al. found that UA could destroy the cell membrane integrity of *Cryptococcus neoformans*, consequently leading to its antifungal effects [31]; Carletto et al. demonstrated that UA could increase dermal collagen synthesis during angiogenesis and thereby improve wound healing [32]. Based on the above research results, UA has extremely high development potential as a natural anti-breast cancer treatment for adjuvant treatment.

It is generally accepted that the low bioavailability of active chemicals is correlated to their poor solubility in water, which limits their applications in the pharmaceutical, food, and cosmetic industries [33,34]. Some physicochemical properties, such as large particle size, small surface area, lipophilic structure and crystalline form, are common to compounds with poor water solubility. [35]. Raw UA, indeed, possesses these physicochemical properties, including large particle size (3370.00 ± 320.07 nm), an irregular particle powder of 2–50 μm, low surface area, and an obvious crystalline form. Chen et al. revealed that UA has very poor water solubility, about 5.6 μg/mL [36]. If these drawbacks could be managed, the activity of UA would be greatly limited. Therefore, UA has also been formulated into many dosage forms to overcome its poor absorption problem.

Nanofibers are a formulation widely used in biomedicine with a high pore volume, a low density and large surface area. They could reduce the volume of control release, increase the stability of active ingredients and oral drugs, and improve bioavailability [37,38]. In this study, UANFs were successfully prepared using HPBCD and PVPK90 as carriers through the process of electrospinning. Improvement in water solubility is the main indicator for determining the best formulation, and our results showed that 1:8:40 UA: PVPK90: HPBCD maintains the highest water solubility. The effect indicated that UANFs effectively improve the water solubility of pure UA, in accordance with the HPBCD ratio. Likewise, higher proportions of cyclodextrin can enhance the encapsulation abilities of formulations, which had also been shown in previous studies [39,40]. In order to recognize the mechanism of improving the water solubility of UA, we compared the physicochemical properties of pure UA and UANFs. The UA nanofibers were uniform nanosized filaments, and the particle size analysis results mentioned that the UANF particles dissolved in water were nano-sized particles with excellent distribution uniformity. These observations indicate that UANFs have a larger surface area than pure UA. Furthermore, the FTIR and ^1^H NMR spectra of UANFs showed that the UA was efficiently encapsulated into excipients, and a stable nanofiber structure was formed through intermolecular hydrogen bonds between the UA and excipients. The formation of intermolecular hydrogen bonds between active compounds and the carriers also improve water solubility. The XRD pattern of UANFs showed that the crystalline structure of the UA transforms into an amorphous structure after forming nanofibers. The conversion of the crystalline to the amorphous form of compounds is also an indicator of improved water solubility; similar results were observed for several active compounds loaded into nanofibers [39,41].

To confirm whether the increase in water solubility indeed increases the skin absorption of UA, the Franz cell diffusion test was conducted. In vitro skin penetration results show that UANFs penetrate the *Stratum corneum* more easily and faster than UA and remain in the epidermis and dermis in large numbers. This outcome confirms that UANFs can significantly improve the absorption of raw UA by the skin. Therefore, if UANFs are used as a dressing for the gold treatment of breast cancer, their effectiveness can be truly demonstrated.

To determine whether UANFs have superior anti-breast cancer activity than raw UA, several observations were made. Our results indicated that UANFs in PBS and raw UA in DMSO treatment effectively inhibited MCF-7 cell growth but this was not displayed in raw UA treatment. These findings demonstrated that after the electrospinning process, UANFs still maintained their anticancer activity on MCF-7 cells. Next, the present study determined the protein expression of MCF-7 cells after raw UA and UANF treatment to verify the anticancer activity. The results showed that UA had no significant effect on the protein expressions of apoptosis. It is speculated that because PBS is used as the solvent for UA, the raw UA has poor water solubility and cannot be dissolved in PBS, so its anticancer activity could not have a significant effect. Therefore, we focused on the anti-breast cancer activity of UANFs. Mitogen-activated protein kinase (MAPKs) is an enzyme that phosphorylates mitogen-activated proteins [23]. The MAPKs signaling pathway regulates cellular functions such as proliferation, survival, and apoptosis [42]. The results showed that UANFs significantly inhibited the phosphorylation of the MAPKs downstream signal ERK1/2 but had no significant effect on the phosphorylation of p38. In recent cancer treatment research, the RAS/RAF/MEK/ERK signaling pathway has become an important target [43]. The central signaling kinase that controls cell survival, apoptosis, and proliferation is the protein kinase ERK1/2. Additionally, the cancer proliferative role of Erk1/2 is a well-established phenomenon [44]. ERK inhibitors have been shown to decrease the survival of multiple human cancer cell lines and the development of melanoma xenografts [45,46]. Previous studies have shown that UA has the effect of inhibiting ERK1/2 phosphorylation, aligning with our results, which also indicated that UANFs express anti-breast cancer effects on ERK-related pathways.

Moreover, the past literature has confirmed that STAT3 plays an important role in the development of various cancers. Dysregulated STAT3 promotes tumorigenesis by altering the expression of genes that regulate cell cycle and cell growth, leading to the disruption of these processes. STAT3 positively regulates cell survival to repress apoptosis, and inversely, STAT3 degradation and inhibition cause increased apoptosis [47]. The increased expression of the downstream apoptotic protein caspase-3 will also promote cell apoptosis. UANFs could also inhibit the expression of STAT3 protein in MCF-7 and significantly upregulate the expression of caspase-3. Thus, it is considered that UANFs could achieve anti-breast cancer effects by promoting the apoptosis of cancer cells. Some studies have pointed out that the combined inhibition of the RAS/RAF/MEK/ERK and STAT3 pathways could have better anti-cancer effects [43]. UANFs could inhibit ERK and STAT3 at the same time, and it is conjectured that UANFs have potential anti-cancer effects.

This study also shows that UANF treatment can effectively inhibit TNF-α expression in MCF-7 cells. TNF-α has pleiotropic effects, and its anticancer activity in animal models may be due to direct cytotoxicity, effects on tumor vasculature, or enhancement of specific anti-tumor immunity. However, other activities of this cytokine could contribute to tumor progression. TNF-α can stimulate angiogenesis and osteoclastic bone resorption and can contribute to cachexia and anemia [24,48]. In cultured breast cancer cells, TNF-α, via the induction of the production of MMP-2 and MMP-9 in tumor-associated macrophages (TAMs), can contribute to the invasion of neoplastic cells into surrounding tissues [25,49]. According to the above research, UANFs may be able to slow down the metastasis of breast cancer cells by inhibiting the expression of TNF-α.

In conclusion, we successfully prepared ursolic acid nanofibers (UANFs) and improved the water solubility of UA by improving physical and chemical properties, including increased surface area, intermolecular bonding with excipients, and amorphous transformation. Moreover, it has also been confirmed that UANFs do have better skin absorption capabilities. In this study, we also confirmed that UANFs have better anti-breast cancer activity. UANFs can indeed inhibit the growth of MCF-7 breast cancer cells, inhibit the expression of phospho-signal transducer and activator of transcription 3 (STAT3) and phospho-extracellular regulated protein kinase (ERK) 1/2, and induce cleaved expression of caspase-3 protein. Consequently, we suggested that UANFs are highly suitable for development as an emerging treatment for breast cancer, and follow-up studies are expected to conduct more detailed efficacy and safety trials to promote the popularization of UANF breast cancer treatment.

## Figures and Tables

**Table 1:** Drug loading, water solubility, encapsulation efficiency, particle size analysis of various UA nanofibers.

Ratio (UA:PVP:HPBCD)	Drug Loading (%)	Solubility (µg/mL)	Encapsulation Efficiency (%)	Particle Size (nm)	Polydispersity Index (PDI)
pure ursolic acid	-	˂LOD *	-	3370.00 ± 320.07	1.43 ± 0.12
1:8:10	98.88 ± 8.44	84.24 ± 10.18	>99	-	-
1:8:20	90.5 ± 6.81	192.17 ± 39.44	>99	-	-
1:8:40	83.97 ± 0.95	606.61 ± 51.91	>99	258.87 ± 14.81	0.29 ± 0.04

* LOD: Limit of detection (<0.01 μg/mL).

## Data Availability

Data for this study are available from the corresponding author upon reasonable request.
